# Association between mental health screening and healthcare utilization among refugees and asylum seekers in Sweden: A retrospective cohort study

**DOI:** 10.1016/j.jmh.2026.100432

**Published:** 2026-07-30

**Authors:** Sara Delilovic, Anna Clara Hollander, Knut Lönnroth, Henna Hasson

**Affiliations:** a Procome Research Group, Department of Learning, Informatics, Management and Ethics, (LIME) Karolinska Institutet (KI) and Centre for Epidemiology and Community Medicine (CES, with Swedish Acronym), Region Stockholm, Stockholm, Sweden; b Epidemiology of Psychiatric Conditions, Substance Use and Social Environment (EPiCSS), Department of Global Public Health, KI, Stockholm, Sweden; c Global Child Health and the Sustainable Development Goals of Global Public Health, Department of Global Public Health, KI, Stockholm, Sweden

**Keywords:** Refugees, Asylum seekers, Utilization, Screening, Primary care, Mental health

## Abstract

**Background:**

Asylum seekers and refugees are at increased risk of mental illness. Previous research suggests underutilization and delayed access to care in this the population. This study aims to explore if screening during a health examination is associated with mental health care utilization among refugees and asylum seekers.

**Methods:**

A retrospective cohort study examined mental healthcare utilization patterns during the five months following voluntary health examinations among asylum seekers and refugees screened with the Refugee Health Screener (RHS-13). We analyzed data from 7726 individuals across five primary healthcare clinics in the Stockholm Region, Sweden, between 2019 and 2022. Outcomes included utilization of mental healthcare within primary care, with and without a recorded diagnosis, and referrals to other mental health units.

**Results:**

Individuals who were screened had a fivefold increase in mental healthcare utilization compared to those who were not screened (odds ratio (OR) 5.09, 95% confidence interval (CI): 3.69–7.04). Both individuals aged 26–39 OR 2.13, (95% CI 1.37–3.31) and 40 year and older OR 2.11, (95% CI 1.34–3.31) were significantly more likely to utilize healthcare compared to the youngest group. Refugees and asylum seekers from the Middle East and North Africa (OR 0.59, 95% CI 0.38–0.90) had a lower likelihood of utilizing mental health care compared to those from Asia.

**Conclusion:**

Screening was associated with a significant increase in mental healthcare utilization. This underscores that integrating mental health screening into routine health examinations can identify mental illness earlier and create more accessible pathways to mental health services.

## Highlights

• Refugees and asylum seekers screened with RHS-13 was five times more likely to utilize mental health care, compared to refugees and asylum seekers in Sweden not screened.
• Age and region of origin was associated with mental health care utilization among both screened and not screened.
• To improve early identification and increase healthcare utilization among refugees and asylum seekers, mental health screening should be integrated into routine health examinations.

## 1 Background

Wars, conflicts, and human rights violations have prompted millions of people to seek protection outside their home countries (United Nations High Commissioner for Refugees 2023). By the end of June 2024, the global population of forcibly displaced people, reached an estimate of 122.6 million. Of these, 72.1 million are internally displaced people, 43.7 million are refugees under international law and 8 million asylum seekers (United Nations High Commissioner for Refugees 2024a) Due to unfavorable developments in major geopolitical crises the United Nations High Commissioner for Refugees (UNHCR) expect forced displacement to increase further throughout the coming years (United Nations High Commissioner for Refugees 2024b).

### 1.1 Mental illness among asylum seekers and refugees

Studies have repeatedly found that asylum seekers and refugees display a higher prevalence of mental health illness and specific conditions such as depression, anxiety, PTSD, and psychosis disorder compared with host country populations and other migrants, although they have lower or similar risk of suicide and substance use disorders (Magwood et al., 2022; Mesa-Vieira et al. 2022; Patanè et al. 2022; Posselt et al. 2020; Termorshuizen and Selten 2023; World Health Organization 2022). This could be attributed to conditions and stressors faced before, during and after migration (Blackmore et al. 2020; Hoell et al. 2021; Magwood et al. 2022; Mesa-Vieira et al. 2022; Uphoff et al. 2020). Despite high burden of mental illness, refugees in high income countries utilize mental health services less and often exhibit different patterns of service use (Brendler-Lindqvist, Norredam, and Hjern 2014; Satinsky et al. 2019) as compared with the native born population. Studies have found that factors contributing to the underutilization of mental health care include limited awareness of available mental health services, delayed help-seeking behaviors and cultural differences in preferences for mental health care services. Mental health care utilization patterns have been shown to vary by region of origin. In Sweden, migrants from sub-Saharan Africa and Asia use less psychiatric care of any sort while migrants from the Middle East and North Africa use more but primary due to psychotropic medication prescriptions (Hollander et al. 2020). Youth refugee and non-refugee migrants in Sweden aged 19–25 use less psychiatric care than Swedish-born youth, except for care related to psychotic disorders. However, utilization rates increased with the length of stay in the host country, eventually aligning with the patterns of the majority population (Björkenstam et al. 2022; Brendler-Lindqvist, Norredam, and Hjern 2014; Satinsky et al. 2019; Straiton et al. 2019)

### 1.2 Referral pathways and early access to mental health services

Despite a comprehensive healthcare system in many host-countries, refugees and asylum seekers face logistical, organizational, as well as linguistic and cultural barriers to mental health services (Lebano et al. 2020; Priebe, Giacco, and El-Nagib 2016; Satinsky et al. 2019). These challenges can aggravate pre-existing health conditions, particularly mental health issues originating from trauma and displacement (Hynie 2018; World Health Organization 2022). Levesque et al.'s (2013) patient-centered framework conceptualizes access to healthcare as a pathway consisting of different stages including having a need, perceiving that need, seeking care, reaching and using services, and receiving appropriate treatment. Barriers arise at each stage from both demand-side (patient) and supply-side (system).

Bridging these barriers and simplifying referral pathways is important to increase timely access to mental health services for refugees and asylum seekers. Integrating mental health screening into routine health assessments may help identify individuals at risk of mental illness early, enabling referrals to appropriate care. The health examination (HE), which is a service offered to asylum seekers and refugees in Sweden, could serve as an entry point in identifying conditions of mental illness, whilst offering appropriate care pathways to mental health care services (Blackmore et al. 2020; Delilovic et al. 2023; Della Rocca et al. 2024). Rather than depending on individuals to recognize symptoms and seek care themselves, early screening within HE can generate and promote demand by proactively identifying needs and initiating referral.

### 1.3 The health examination for asylum seekers and refugees in Sweden

The refugee situation in Sweden has undergone significant changes in recent years. Following the large influx of asylum seekers in 2015–2016, Sweden implemented stricter immigration policies with temporary residence permits becoming the norm rather than permanent ones. Healthcare for refugees and asylum seekers in Sweden is primarily regulated through the Act on Health and Medical Care for Asylum Seekers, which grants adult asylum seekers the right to "care that cannot be deferred," maternity care, contraceptive counseling, and abortion services (Government Offices of Sweden, 2008). Quota refugees are entitled the same health care as permanent residents.

In Sweden, all asylum seekers and refugees have the opportunity to undergo a voluntary HE, free of charge at primary health care clinics. The objective of HE is to assess mental and physical healthcare needs, requiring immediate attention, including standardized screening for selected infectious diseases. Participation in all components of the assessment is voluntary. The HE has up until now lacked standardized procedures for addressing mental illness in Stockholm Region. In Stockholm Region health care professionals have used different medical checklists or templates when conducting the examination, leading to varied practices both within but also between health care clinics.

### 1.4 Screening measurement for mental illness among refugees

*The Refugee Health Screener RHS-13 is* designed to evaluate symptoms related to common mental health issues among refugees, including post-traumatic stress disorder (PTSD), depression, and anxiety. The original version of the instrument, consisting of 15 items, demonstrated excellent psychometric properties, with high internal consistency (Cronbach's α = 0.91) (Hollifield et al. 2013, 2016a). A shorter 13-item version of the RHS was later developed, which maintained strong psychometric properties (Cronbach's α = 0.96) without compromising the validity. Respondents complete the RHS-13 using a five-point Likert scale, with scores ranging from 0 (“Not at all”) to 4 (“Extremely”), producing a total score between 0 and 52. A cut-off score of ≥11 has been recommended to identify positive screenings for mental illness. Studies have categorized cut-offs for symptom severity as mild (11–17), moderate (18–24), or severe (≥25) (Bjärtå et al. 2018; Hollifield et al. 2016b). The psychometric properties of the 13-item RHS have been established in the Swedish context, in a study among asylum seekers from Afghanistan, Syria, Iraq, Iran, Somalia, and Eritrea reporting good internal consistency (Cronbach's α = 0.92) (Bjärtå et al. 2018).

This study aims to explore if screening with The Refugee Health Screener (RHS-13) during the HE is associated with mental health care utilization among refugees and asylum seekers and if it differs by sex, legal status, age and region of origin.

## 2 Material and methods

### 2.1 Study design and setting

#### 2.1.1 Setting

Prior to this study, RHS-13 was implemented and integrated into the HE during 2019–2021 in selected primary health clinics in Stockholm Region. Introduction was completed by a nurse with prior experience in HEs. These included: one educational meeting covering RHS-13 administration, score interpretation, referral procedures/recommendations for different outcome scores, mental illness prevalence among forced migrants, documentation practices and distribution of educational materials. A biannual learning collaborative was implemented within an existing local network to facilitate continuous peer learning and ongoing implementation support. The use of RHS-13 was recommended to health professionals, but not compulsory.

### 2.2 Data collection

This retrospective cohort study was conducted in the Stockholm Region, Sweden, using register-based data. Routine health data were collected from the electronic health records (EHR) and included data from five primary healthcare clinics offering HEs to asylum seekers and refugees. Data was pseudonymized and include demographic data and data on health care utilization for the period from April 1st, 2019, until June 30th, 2022. All individuals aged 14 or older who were eligible for the health examination were included in the study.

### 2.3 Variables

#### 2.3.1 Exposure

Our primary exposure was completion of screening with RHS-13, categorized as completed or not completed. It accounts for if screening with RHS-13 was completed during the HE. Reasons for non-screening with RHS-13 were not recorded.

#### 2.3.2 Outcome(s)

Healthcare utilization was assessed during a five-month period following the HE. Only healthcare contacts that occurred after the completion of the examination were included, ensuring a temporal sequence in which exposure (screening) preceded outcome (utilization). This approach was to capture utilization that may have been influenced by the screening process. Same day contacts are included. All outcomes examined in the analysis are formally accessible to all participants, including asylum seekers, under Swedish healthcare policy.

Mental healthcare utilization was defined as any of the following:

**Mental healthcare within primary care**: defined as a visit within primary care with a nurse, physician, psychologist, or psychotherapist where the encounter was registered under a mental health service mandate. Healthcare encounters in Region Stockholm are coded using Kombika (in Swedish) (combined facility-clinic-department codes), which identify care delivery units at the most granular administrative level. Primary health care clinics are assigned multiple facility codes corresponding to distinct service mandates: general primary care, basic home healthcare, and mental health services. By looking at the Kombika under which a healthcare contact is registered, it is possible to determine which service it belongs to.

**Mental healthcare for mental or behavioral disorders**: defined as a visit within primary care with a physician resulting in an ICD-10 (International Statistical Classification of Diseases and Related Health Problems, 10th Revision) diagnosis within the F00–F99 category (World Health Organization. 2011). The dataset included only confirmed visits with a set diagnosis; the specific diagnosis was not available.

**Referral to other unit for mental illness**: Three data fields were examined for referrals: the receiving healthcare provider or department, the stated consultation reason, and the diagnosis or clinical question specified by the referring clinician. Referrals were coded as mental health-related if any of these fields indicated psychological, psychiatric, or mental health concerns. Visits could not be confirmed through available records, only the sent referral.

**Any utilization** indicates whether any of these forms of utilization occurred.

#### 2.3.3 Other variables

**Sex:** Sex assigned at birth categorized into men and women.

**Age:** was categorized into the following groups: 14–25 years, 26–39 years and 40 years and older

**Region of origin:** Region of origin was determined based on country of birth, organized into five main regions: Asia, Europe and Eastern Europe, the Middle East and North Africa, Sub-Saharan Africa, and Americas.

**Legal status:** Legal status, classified as "asylum seekers" or "quota and other refugees." The "other" category included people who entered Sweden through family reunification, with eligibility for a residence permit prior to arriving in Sweden due to having one or more family members who had been granted refugee status or subsidiary protection (i.e., individuals who do not qualify as refugees but would face a significant risk of serious harm if returned to their home country). Those with family reunification status were categorized into the "quota and others" group, as they were expected to share similar characteristics and legal benefits with quota refugees.

### 2.4 Statistical analysis

The statistical analysis was conducted to examine the association between exposures and the likelihood of the outcome(s). Categorical variables were presented as frequencies and percentages, and differences were assessed using chi-square tests of independence. Logistic regression estimated crude odds ratios (OR) for associations between exposure variables and outcomes. A multivariable model adjusted for confounders (age, sex, region of origin) and provided adjusted odds ratios (AOR) with 95% confidence intervals (CI). Models excluding region of origin were based on the full cohort (n = 7726), whereas Model 2 was restricted to individuals with complete data on this covariate (n = 4849), owing to missing data on region of origin. No interactions were identified among independent variables. Statistical significance was set at p < 0.05, and model fit was assessed using the Hosmer-Lemeshow test. The outcome variable(s) was dichotomized as “utilized healthcare” vs. “not utilized healthcare”. The primary independent variable was completion of screening with RHS-13. All analyses were performed using R and JAMOVI.

Additionally, we did not conduct reliability analyses in the present study, as the validation sample used by Bjärtå et al. (2018) was highly comparable to our population in terms of countries of origin and data collection timeframe.

## 3 Ethical considerations

Ethical approval was granted by the Swedish Ethical Review Authority (Dnr: 2019–01408) All data were pseudonymized and provided by The Health and Medical Care Administration in Stockholm Region. Data recorded during the HE is handled according to Swedish law, as for all citizens. Participation is voluntary, and patients choose what health-related information to share.

## 4 Results

### 4.1 Characteristics of the sample

During the study period, 7726 HEs were conducted, with 532 (6.9%) completing RHS-13 screening. The majority were asylum seekers (84.4%). Among 4849 individuals with recorded country of origin, the largest groups were Uzbeks, Afghanis, Syrians, Ukrainians, Mongolians, and Eritreans. Regionally, most participants came from Sub-Saharan Africa (32%) and the Middle East and North Africa (31.1%) (Table 1). Among those who completed screening, 333 individuals (62.6%) screened negative (RHS-13 score 0–10), while 199 (37.4%) screened positive (score ≥11), indicating probable mental illness. Among those with a positive screen, 52 (9.8% of the total sample) reported mild symptoms (11–17), 47 (8.8%) reported moderate, clinically significant symptoms (18–24), and 100 (18.8%) reported severe or acute symptoms (≥25) (data not shown in tables).

**Table 1:** Sample Characteristics of respondents.

Sample Characteristics		
**Total**	N = 7726	
	n	(%)
**Clinic**		
Clinic 1	1384	17.9
Clinic 2	1822	23.6
Clinic 3	2405	31.1
Clinic 4	521	6.7
Clinic 5	1594	20.6
**Completion of screening with RHS-13**		
Yes	532	6.9
No	7194	93.1
**Sex**		
Men	4446	57.5
Women	3280	42.5
**Legal Status**		
Asylum seekers	6518	84.4
Quota and other refugees	1208	15.6
**Age**		
14–25	1503	19.5
26–39	3430	44.5
40>	2793	36.2
**Country of origin (top 6)**		
Afghanistan	426	5.5
Eritrea	243	3.1
Mongolia	262	3.4
Syria	419	5.4
Ukraine	421	5.4
Uzbekistan	531	6.9
**Region of origin**⁎		
Asia	571	11.8
Europe and Eastern Europe	452	9.3
Middle east north Africa	1508	31.1
Sub-Sahara Africa	1553	32.0
Americas	765	15.8

⁎ N = 4849 due to missing data on region of origin.

### 4.2 Factors associated with mental health care utilization

Screening with the RHS-13 was significantly associated with higher overall healthcare utilization (Table 2). Screened individuals were more likely to utilize any healthcare services compared to non-completers (p < 0.01). Referrals to other clinics were also more frequent among screened individuals than non-screened (p < 0.01). Asylum seekers exhibited higher healthcare utilization compared to quota and other refugees (p < 0.01). They also had significantly higher healthcare contact rates for mental and behavioral disorders (p < 0.01). Age influenced healthcare utilization, with individuals aged 26–39 having higher rates compared to those aged 14–25 (p < 0.01). Similar age patterns were observed for the mental health care contacts in primary care (p = 0.007) and referrals to other clinics (p = 0.032). No significant differences were found by region of origin, except for referrals to other units (p = 0.015) with individuals from the Americas, having nearly four times higher rates than those from Europe and Eastern Europe. Sex differences were minimal, with no significant variations in healthcare utilization across outcomes.

**Table 2:** Prevalence (%) of health care utilization for outcome(s): Any utilization; Mental healthcare within primary care; Mental healthcare for mental or behavioral disorders and Referral to other unit for mental illness; by completion of screening with RHS-13, sex, legal status, age, and region of origin.

**Health Care Utilization**								
	Any utilization	No health care utilization	Mental healthcare within primary care	No mental healthcare within primary care	Mental healthcare for mental or behavioral disorders	No mental healthcare for mental or behavioral disorders	Referral to other unit for mental illness:	No referral to other unit for mental illness
	**n=341**	**n=7358**	**n= 128**	**n =7598**	**n= 74**	**n=7652**	**n=197**	**N=7529**
**Total (%)**	4.5	95.5	1.7	98.3	1.0	99.0	2.5	97.5
	**% (n)**	**% (n)**	**% (n)**	**% (n)**	**% (n)**	**% (n)**	**% (n)**	**% (n)**
**Completion of screening with RHS-15**								
Yes	13.7 (73)	86.3 (459)	6.0 (32)	94.0 (500)	0.9 (5)	99.1 (527)	9.0 (48)	91.0 (484)
No	3.7 (268)	96.3 (6926)	1.3 (96)	98.7 (7098)	1.0 (69)	99.0 (7125)	2.1 (149)	97.9 (7045)
**Sex**								
Men	4.2 (185)	95.8 (4261)	1.6 (73)	98.4 (4373)	0.9 (40)	99.1 (4406)	2.3 (103)	97.7 (4343)
Women	4.8 (156)	95.2 (3124)	1.7 (55)	98.3 (3225)	1.0 (34)	99.0 (3246)	2.9 (94)	97.1 (3186
**Legal Status**								
Asylum seeker	4.8 (313)	95.2 (6205)	2.0 (128)	98.0 (6390)	1.1 (73)	98.9 (6445)	2.6 (169)	97.4 (6349)
Quota and other refugees	2.3 (28)	97.7 (1180)	0.0 (0)	100.0 (1208)	0.1 (1)	99.9 (1207)	2.3 (28)	97.7 (1180)
**Age**								
14–25	2.9 (43)	97.1 (1460)	0.7 (11)	99.3 (1492)	0.9 (13)	99.1 (1490)	1.9 (29)	98.1 (1474)
26–39	5.0 (170)	95.0 (3260	1.9 (11)	98.1 (3366)	0.8 (29)	99.2 (3401)	3.1 (105)	96.9 (3325)
40≥	4.6 (128)	95.4 (2665)	1.9 (53)	98.1 (2740)	1.1 (32)	98.9 (2761)	2.3 (63)	97.7 (2730)
**Region of origin**								
Asia	6.7 (38)	93.3 (533)	2.8 (16)	97.2 (555)	1.1 (6)	98.9 (565)	3.7 (21)	96.3 (550)
Europe and Eastern Europe	3.5 (16)	96.5 (436)	1.8 (8)	98.2 (444)	0.7 (3)	99.3 (449)	1.3 (6)	98.7 (446)
Middle east north Africa	4.2 (63)	95.8 (1445)	1.8 (27)	98.2 (1481)	0.9 (14)	99.1 (1494)	2.1 (32)	97.9 (1476)
Sub-Sahara Africa	5.3 (83)	94.7 (1470)	1.4 (22)	98.2 (1481)	1.5 (23)	98.5 (1530)	3.3 (52)	96.7 (1501)
Americas	5.2 (40)	94.8 (725)	1.6 (12)	98.4 (753)	0.5 (4)	99.5 (761)	3.9 (30)	96.1 (735)

### 4.3 Unadjusted and adjusted models for mental healthcare utilization

#### 4.3.1 Any health care utilization

Completion of screening with RHS-13 was significantly associated with increased likelihood of healthcare utilization across all outcomes. Individuals who completed the screening had 4.11 times higher odds of using any healthcare services (95% CI: 3.12–5.41) compared to non-completers. This association remained in adjusted models: 4.02 (95% CI: 3.05–5.31) after adjusting for age and sex (Goodness-of-fit test: χ² = 0.47, df = 4, p = 0.97), and 5.09 (95% CI: 3.69–7.04) (Goodness-of-fit test: χ² = 6.95, df = 8, p = 0.54) when also adjusting for region of origin (Table 3). Age was significantly associated with any healthcare utilization. Crude analyses showed that individuals aged 26–39 had 1.77 times higher odds of utilizing any healthcare (95% CI: 1.26–2.48) compared to the youngest group (14–25). Similarly, individuals in the oldest age group had 1.63 times higher odds (95% CI: 1.14–2.31). In the adjusted model, the odds for the 26–39 group were 1.69 (95% CI: 1.20–2.38), and for the 40 and older group, they were 1.62 (95% CI: 1.13–2.30). In the fully adjusted model, the odds increased to 2.13 (95% CI: 1.37–3.31) for the 26–39 group and 2.11 (95% CI: 1.34–3.31) for the oldest group. Region of origin showed significant associations with any healthcare utilization both in crude and adjusted models. Fully adjusted models showed, individuals from the Middle East and North Africa had significantly lower odds of utilization compared to those from Asia (OR: 0.59 CI 0.38- 0.90) No significant differences were observed for sex, where odds of healthcare utilization remained similar for men and women across all outcomes.

**Table 3:** Crude odds ratios (OR) and adjusted odds ratios (AOR) for the associations between Any health care utilizations and completion of screening with RHS-13, sex, legal status, age, region of and origin with 95% confidence intervals (CI), for total sample.

**Any health care utilization**			
N = 341			
	Crude OR	Model 1	Model 2
	**OR (95% CI)**	**AOR (95% CI)**	**AOR (95% CI)**
**Completion of screening with RHS-13**			
No	**1**	**1**	**1**
Yes	4.11(3.12–5.41) ^⁎⁎^	4.02 (3.05–5.31)^⁎^	5.09 (3.69–7.04)^⁎^
**Sex**			
Men	**1**	**1**	**1**
Women	1.15 (0.92–1.43)	1.15 (0.92–1.44)	1.09 (0.84–1.43)
**Legal Status**			
Quota and other refugees	**1**	**-**	**-**
Asylum seekers	2.12 (1.43–3.14)^⁎⁎^	-	-
**Age**			
14–25	**1**	**1**	**1**
26–39	1.77 (1.26–2.48)^⁎⁎^	1.69 (1.20–2.38)^⁎⁎^	2.13 (1.37–3.31)^⁎^
40≥	1.63 (1.14–2.31)^⁎^	1.62 (1.13–2.30)^⁎⁎^	2.11 (1.34–3–31)^⁎^
**Region of origin**			
Asia	**1**		**1**
Europe and Eastern Europe	0.51 (0.28–0.93)^⁎^		0.55 (0.30–1.01)
Middle east north Africa	0.61 (0.40–0.92)^⁎^		0.59 (0.38–0.90)^⁎^
Sub-Sahara Africa	0.79 (0.53–1.17)		0.98 (0.65–1.48)
Americas	0.77 (0.48–1.22)		0.99 (0.64–1.59)

*p value < 0.05, **p-value < 0.01, ***p-value < 0.00. *Note.* Crude ORs are based on the full sample (n = 7726), except region of origin (n = 4849). Model 1 (excluding region of origin) uses the full sample; Model 2 (including region of origin) uses the complete-case sample (n = 4849).

#### 4.3.2 Mental healthcare within primary care

Individuals who completed screening had 4.73 times higher crude odds (95% CI: 3.14–5.03) compared to non-completers (Table 4). After adjusting for age and sex, the odds slightly increased to 4.63 (95% CI: 3.07–6.99) (Goodness-of-fit test: χ² = 0.59, df = 1, p = 0.43).In the fully adjusted model, the odds rose further to 5.13 (95% CI: 3.13–8.41) (Goodness-of-fit test: χ² = 1.46, df = 8, p = 0.99). No significant differences were found for gender in crude or adjusted models. Regarding age, individuals aged 26–39 had significantly higher odds of using mental healthcare within primary care (OR: 2.57, 95% CI: 1.35–4.90) compared to the youngest group (14–25). Similarly, those aged 40 and older showed significantly increased odds (OR: 2.62, 95% CI: 1.36–5.03). The association were evident in the model 1, with increased odds for higher age 26–39 (OR = 2.41, CI 1.26–4.60), 40 and older (OR=2.59, CI 1.38–4.98). Age was only significant for the oldest group in the fully adjusted model (2.38, CI 1.09–5.18). No association was found for country of origin, except in the unadjusted model, whereas individuals from Sub-Saharan Africa had 49% lower odds of utilizing healthcare compared to the reference group (OR = 0.49, CI 0.25–0.95).

**Table 4:** . Crude odds ratios (OR) and adjusted odds ratios (AOR) for the associations between Mental healthcare within primary care and completion of screening with RHS-13, sex, legal status, age, regions of origin with 95% confidence intervals (CI), for total sample.

**Mental healthcare within primary care**			
N = 128			
	Crude OR	Model 1	Model 2
	**OR (95% CI)**	**AOR (95% CI)**	**AOR (95% CI)**
**Completion of screening with RHS-13**			
No	**1**	**1**	**1**
Yes	4.73 (1.36–5.03)^⁎⁎^	4.63 (3.07–6.99)^⁎^	5.13 (3.13–8.41)^⁎^
**Sex**			
Men	**1**	**1**	**1**
Women	1.02 (0.71–1.45)	1.02 (0.72–1.46)	1.00 (0.64–1.56)
**Legal status**			
Quota and other refugees	**1**	**-**	**-**
Asylum seekers	-^⁎⁎⁎^	-	-
**Age**			
14–25	**1**	**1**	**1**
26–39	2.57 (1.35–4.90)^⁎⁎^	2.41 (1.26–4.60)^⁎⁎^	1.98 (0.91–4.31)
40≥	2.62 (1.36–5.03)^⁎⁎^	2.59 (1.34–4.98)^⁎⁎^	2.38 (1.09–5.18)^⁎^
**Region of origin**			
Asia	**1**		**1**
Europe and Eastern Europe	0.62 (0.26–1.47)		0.68 (0.28–1.61)
Middle east north Africa	0.63 (0.33–1.18)		0.60 (0.32–1.14)
Sub-Sahara Africa	0.49 (0.25–0.95)*		0.61 (0.31–1.19)
Americas	0.55 (0.25–1.17)		0.71 (0.33–1.53)

*p value < 0.05, **p-value < 0.01, ***p-value < 0.00. *Note.* Crude ORs are based on the full sample (n = 7726), except region of origin (n = 4849). Model 1 (excluding region of origin) uses the full sample; Model 2 (including region of origin) uses the complete-case sample (n = 4849).

#### 4.3.3 Mental healthcare for mental or behavioral disorders

No statistically significant differences in healthcare contact for mental disorders and behavioral disorders (ICD 10 F00-F99) were identified based on completion of RHS-13, gender, legal status, age, or region of origin in unadjusted or adjusted models (Table 5).

**Table 5:** Crude odds ratios (OR) and adjusted odds ratios (AOR) for the associations between Mental healthcare for mental or behavioral disorders (ICD 10 F00-F99) and completion of screening with RHS-13, sex, legal status, age, regions of origin with 95% confidence intervals (CI), for total sample.

**Mental healthcare for mental or behavioral disorders**			
N = 74			
	Crude OR	Model 1	Model 2
	**OR (95% CI)**	**AOR (95% CI)**	**AOR (95% CI)**
**Completion of screening with RHS-13**			
No	**1**	**1**	**1**
Yes	0.98 (0.39–3.02)	0.52 (0.12–2.16)	0.53 (0.12–2.23)
**Sex**			
Men	**1**	**1**	**1**
Women	1.15 (0.72–1.82)	1.02 (0.58–1.79)	1.02 (0.58–1.80)
**Legal status**			
Quota and other refugees	**1**	**-**	**-**
Asylum seekers	-^⁎⁎⁎^	-	-
**Age**			
14–25	**1**	**1**	**1**
26–39	0.97 (0.50–1.88)	1.35 (0.57–3.20)	1.43 (0.59–3.43)
40≥	1.32 (0.64–2.53)	1.66 (0.71–3.92)	1.73 (0.73–4.13)
**Region of origin**			
Asia	**1**		**1**
Europe and Eastern Europe	0.62 (0.15–2.30)		0.61 (0.15–2.45)
Middle east north Africa	0.88 (0.33–2.30)		0.87 (0.33–2.28)
Sub-Sahara Africa	1.41 (0.57–3.49)		1.44 (0.58–3.57)
Americas	0.49 (0.13–1.76)		0.51 (0.14–1.83)

*p value < 0.05, **p-value < 0.01, ***p-value < 0.00. *Note.* Crude ORs are based on the full sample (n = 7726), except region of origin (n = 4849). Model 1 (excluding region of origin) uses the full sample; Model 2 (including region of origin) uses the complete-case sample (n = 4849).

#### 4.3.4 Referral to other unit for mental illness

Crude analysis found individuals completing screening had 4.68 times higher odds of being referred to other units (95% CI: 3.34–6.57) compared to non-completers (Table 6). Both models found completion of screening to be associated with referrals, model 1 with adjustment for age and sex (OR = 4.55 CI 3.24–6.38) (Goodness of fit test; χ2 = 1.75, df= (4), p = 0.78) and model 2 (OR = 6.0 CI 4.04–8.92) (Goodness of fit test; χ2 = 4.04, df= (8), p = 0.85). Individuals in age group 26–39 (OR = 2.39 CI 1.37–4.16) where more likely to have a referral to other unit for mental illness, in opposite to their referees (14–25) for unadjusted and adjusted models, (OR = 2.39 CI 1.37–4.16) AOR model 1 (OR = 1.52 CI 1.00–2.32) and AOR model 2, (OR = 2.39 CI 1.37–4.16). When examining region of origin, individuals from Europe and Eastern Europe had significantly lower odds of being referred to other units compared to those from Asia (OR: 0.35, 95% CI: 0.14–0.88). Equally, individuals from the Middle East and North Africa also exhibited lower odds of referral (OR: 0.56, 95% CI: 0.32–0.99). Adjusted model indicated similar patterns where individuals from Europe and eastern Europe, Middle East and North Africa had slightly lower odds of being referred to other units compared to individuals originating from the Asian region (OR = 0.39 CI 0.15–0.99, OR = 0.55 CI 0.31–0.99, respectively).

**Table 6:** Crude odds ratios (OR) and adjusted odds ratios (AOR) for the associations between Referral to other unit for mental illness and completion of screening with RHS-13, sex, legal status, age, regions of origin with 95% confidence intervals (CI), for total sample.

**Referral to other unit for mental illness**			
N = 197			
	Crude OR	Model 1	Model 2
	**OR (95% CI)**	**AOR (95% CI)**	**AOR (95% CI)**
**Completion of screening with RHS-13**			
No	**1**	**1**	**1**
Yes	4.68 (3.34–6.57)^⁎⁎^	4.55 (3.24–6.38)*	6.00 (4.04–8.92)*
**Sex**			
Men	**1**	**1**	**1**
Women	1.24 (0.93–1.65)	1.25 (0.94–1.67)	1.18 (0.83–1.66)
**Legal status**			
Quota and other refugees	**1**	**-**	**-**
Asylum seekers	-^⁎⁎⁎^	-	-
**Age**			
14–25	**1**	**1**	**1**
26–39	1.60 (1.05–2.43)^⁎^	1.52 (1.00–2.32)^⁎^	2.39 (1.37–4.16)^⁎^
40>	1.17 (0.75–1.82)	1.16 (0.74–1.81)	1.74 (0.97–3.13)
**Region of origin**			
Asia	**1**		**1**
Europe and Eastern Europe	0.35 (0.14–0.88)^⁎^		0.39 (0.15–0.99)^⁎^
Middle east north Africa	0.56 (0.32–0.99)^⁎^		0.55 (0.31–0.99)^⁎^
Sub-Sahara Africa	0.90 (0.54–1.52)		1.19 (0.69–2.02)
Americas	1.06 (0.60–1.88)		1.45 (0.81–2.61)

*p value < 0.05, **p-value < 0.01, p-value < 0.00. *Note.* Crude ORs are based on the full sample (n = 7726), except region of origin (n = 4849). Model 1 (excluding region of origin) uses the full sample; Model 2 (including region of origin) uses the complete-case sample (n = 4849).

## 5 Discussion

The aim of the study was to explore if screening with RHS-13 during the HE was associated with mental health care utilization among refugees and asylum seekers, and whether sex, age, legal status and region of origin could explain any differences. The findings support this, showing that screening was associated with a higher likelihood of utilization. Prior studies using the RHS-13 in various contexts have primarily focused on outcomes of the screening. This study adds a novel contribution by demonstrating an association between screening and a higher likelihood of mental healthcare utilization Those who completed screening were nearly five times more likely to utilize health services than those who did not. This is an important finding since evidence points towards both underutilization and delayed access to health care for the group. We also found age and region of origin, beside screening with RHS-13, to be associated with health care utilization, with people from Europe and Eastern Europe and Middle east north Africa being less likely to utilize care compared to their counterparts. Notably, we found no association between health care utilization and legal status. For the differences by region of origin, this could be explained by the varying levels of conflict exposure, pre-migration trauma/experiences and cultural perceptions of mental illness (Delilovic et al. 2023; Duggal et al. 2019; Dykxhoorn et al. 2019; Schlaudt et al. 2020). Additionally, being older was associated with higher health care utilization and these differences might be explained by the increased vulnerability among older whereas younger might be more resilient or less impacted by the prolonged stresses of displacement (Porter and Haslam 2005; Schlaudt et al. 2020; Strømme et al. 2020).

### 5.1 Mental health screening and utilization: impact and challenges

Several studies have shown refugees and other migrants face lower access to outpatient psychiatric services compared to majority populations (Abebe, Lien, and Elstad 2017; Barnett et al. 2019; Hollander et al. 2020; Lindert et al. 2008; Terhune et al. 2022). Identifying mental illness among this group is important as undiagnosed mental health conditions can have a significant impact on employment, integration and resettlement in host-country. Reasons for why screening was associated with higher rates of mental health care utilization in this study can be explained through several interconnected mechanisms.

In our study, healthcare professionals may have played an important role in increasing use of health care services after screening. Using the RHS-13 may have added a more objective assessment, rather than relying solely on clinical judgment and personal experience. By implementing systematic screening, providers might develop awareness of common mental health issues among the group and become more proactive and motivated in addressing as well as offering healthcare services related to mental illness (Magwood et al. 2022; Robertshaw, Dhesi, and Jones 2017). Identifying mental illness might also lead to accountability for healthcare professionals to provide and offer follow-up and leading to more health care utilization for mental illness. This relationship likely operates as a bidirectional process: screening leads to increased referrals to health services while simultaneously empowering individuals to seek healthcare in response to their mental health concerns. However, in our study, we also observed that asylum seekers and refugees who were not screened with RHS-13 still utilized mental health care services, though screening was associated with greater utilization. This could be related to individuals disclosing mental health concerns during the HE, or to healthcare professionals’ ability to identify these concerns without formal screening tools.

Other reasons for the association between mental health care utilization and screening could be that screening may function as a form of psychoeducation, helping refugees and asylum seekers recognize and acknowledging their experiences and mental health concerns. The screening process may have validated patients’ experiences, implying that their symptoms justify mental health services. Furthermore, introducing mental health screening in an already existing procedures as the HE can reduce stigma-related barrier faced by the population leading to more health care utilization, as well as dissolve issues with manifestation of symptoms (Derr 2016; DeSa et al. 2022; Parajuli and Horey 2020; Peterson et al. 2020). This is particularly important for mental illness, where symptom can be culturally influenced or subtle, like when psychological distress manifests through physical symptoms such as headaches, back pain or fatigue etc., a pattern that has been commonly observed within this group (Peñuela-O’Brien et al. 2023; Robertshaw, Dhesi, and Jones 2017). Screening procedures can facilitate identification of mental illness, which might otherwise go undetected, particularly in populations with limited mental health literacy.

Screening in general presumes the condition or disease being screened should have serious health consequences and be preventable or treatable (Wilson and Jungner 1968). Evidence points towards high prevalence of mental health conditions among refugees thus making screening for mental illness applicable, appropriate and justifiable for the population (Blackmore et al. 2020).

Despite its potential benefits, screening for mental illness among refugees and asylum seekers presents significant ethical dilemmas when access to mental health services is limited, potentially identifying needs that cannot be adequately addressed. This is particularly concerning in systems where healthcare eligibility is restricted for certain migrant groups or when pathways to care are inadequate. In Sweden, asylum seekers are entitled only to "care that cannot be postponed," which may restrict access to mental health services regardless of need. The interpretation of "non-postponable care" involves clinical judgment and might vary across providers and settings. We cannot determine from our data how healthcare professionals apply these criteria or how asylum seekers understand their entitlements. The observed low utilization among asylum seekers likely reflects a combination of interacting factors, including providers’ interpretation and application of formal eligibility criteria, as well as individual-level barriers such as system navigation capacity, language barriers, and underlying health needs. Though our data did not allow us to disentangle their relative contributions. Ethical challenges also arise from balancing the benefits of early identification of mental health illness with the risks of stigmatization, re-traumatization, and unintended harm (Davidson, Hammarberg, and Fisher 2024; Seagle et al. 2020).

### 5.2 Barriers to screening participation

A notable finding in our study is the overall low participation in the screening (6.9%). Low screening participation can be attributed to multiple factors related to both healthcare providers and screening recipients. On provider level, limited resources and time constrains can hinder their ability to conduct mental health screenings (Hagström et al. 2024; Peñuela-O’Brien et al. 2023; Robertshaw, Dhesi, and Jones 2017). In settings with high number of patient visits, mental health screening may be deprioritized in favor of addressing other health concerns. Inadequate training and lack of confidence can lead to hesitancy to use screening during the HE, as well as lack of skills in working with culturally diverse populations (Magwood et al. 2022; Peñuela-O’Brien et al. 2023; Robertshaw, Dhesi, and Jones 2017). On individual level, language and communications barrier can hinder participation, particularly when screening is not available in the preferred language or when interpretation are inadequate (Peñuela-O’Brien et al. 2023). Structural and individual barriers faced by refugees and asylum seekers can lead to delayed or missed diagnoses (Satinsky et al. 2019). As a result, even when mental health needs exist, they may not translate into documented healthcare contacts, which could explain the low prevalence of the outcome "Mental healthcare for mental or behavioral disorders" in our study (1%). Due to small subgroup sizes and limited statistical power, we were unable to detect statistically significant associations. These results should be interpreted cautiously, as they may reflect insufficient power, service underutilization, or both, rather than absence of true associations.

### 5.3 Methodological considerations

#### 5.3.1 Strengths and limitation

This study on healthcare utilization among refugees and asylum seekers has several methodological limitations. Firstly, we did not assess the association between RHS-13 score/outcomes and utilization, but rather focused on whether healthcare was utilized based on completion of screening. We were interested in examining whether the act of screening was associated with health care utilization, regardless of individual symptom severity. Another limitation is the timing of screening. Timing combined with context under which the screening is performed is important when assessing the mental health status of asylum seekers and other refugees. Mental health is not a static, but rather a dynamic state of well-being. Post migration factor faced in the host country pose another risk, elevated or more evident after a certain time. Assessment of mental illness in an early phase may lead to an overestimation (or under) of mental health disorders, as initial emotional distress could be a response to post-migration stressors rather than an underlying mental illness. Completing screening during the HE at different times points might have yielded other outcomes and we were not able to control for time-varying component. Also, the relationship between mental health screening and healthcare utilization presents potential reversed causation since individuals with pre-existing mental health illness may be more likely to be offered and participate in screening. Health care providers might be more likely to use screening with refugees who display and present signs of mental illness and refrain from using among those who do not. We had no baseline data on mental health status or past healthcare use, and therefore not able to adjust for pre-existing factors that could have influenced either screening participation or subsequent health care utilization.

Regarding referrals, our inability to track whether patients attended appointments at other healthcare units is a limitation. Many refugees and asylum seekers face disruptions in continuity of care, whether due to difficulties in navigation in the healthcare system, financial barriers, or other structural, linguistic and cultural barriers. However, referrals represent an important step in the care pathway. In Swedish primary care, referrals are necessary gateways to specialized mental health services. Referral generation indicates that an individual: (1) accessed primary care, (2) had mental health concerns identified by a provider, and (3) was formally connected to pathways for specialized care. We acknowledge that referral generation does not guarantee subsequent healthcare utilization, as various barriers may prevent follow-through. However, referrals reflect meaningful engagement between providers and patients, indicating both recognition of clinical need and initiation of care pathways.

Another limitation is the possibility of selection bias. Since the HE was voluntary, individuals who chose not to participate may differ systematically in their mental health status from those who attended, potentially affecting the generalizability of our findings. Another notable limitation is that region of origin was missing for 37.2% of the sample, which reduced statistical power for analyses involving this variable.

We also acknowledge unmeasured confounding may arise from clinic-level factors (quality of mental health services, implementation intensity), provider-level variation (clinician engagement with mental health care), and temporal trends (progressive adoption and increased awareness throughout the study period). These factors may independently influence both screening practices and utilization patterns, and our analyses did not account for such clustering or time-varying effects.

A strength of the study was that data collection was carried out by healthcare professionals within the clinical setting, eliminating the need for outreach methods. We also used a validated screening instrument specifically designed for refugee populations, ensuring reliability and relevance. While conducted in a single region of Sweden, the study included five health care center and data was collected over a three-year period, including a large population of refugees and asylum seekers.

## 6 Implications for practice and policy

The study entails some important practice and policy implications. As the HE is usually the main entrance of health care for asylum seekers and refugees, role of primary care becomes vital in functioning as a gateway to specialized service (Blackmore et al. 2020; Magwood et al. 2022, 2023; Steel et al. 2006). Incorporating mental health screening into already established HEs can be of benefit. HEs in general tend to primarily focus on infectious diseases and often overlook mental health concerns, despite the high prevalence of mental illness in the population (Delilovic et al. 2018). Adding a structured mental health screening like the RHS-13 could provide systematic and standardized approach to early identification of mental illness, ensuring that displaced people receive timely support and referrals for further care. However, it is important to recognize that screening for mental illness only yields benefits when embedded within a care system that offers pathways to health care services. Such an organization requires engagement and coordination to maintain continuity of care, appropriate follow-up and equal access. In addition, this should be delivered using a culturally sensitive approach (Jensen et al. 2013; Mangrio and Sjögren Forss 2017). Currently, mental health services are often inconsistent and unstructured, varying both within and between health care practitioners/health care centers (Delilovic et al. 2018; Hagström et al. 2024). By embedding screening into the HE, refugees, and asylum seekers, regardless of their location, could receive equal opportunity for assessment of mental illness. This approach aligns with the principle of equal access to healthcare, reducing disparities in mental health service utilization. Implementation in the Swedish context should be considered as a potential way forward, and studies have shown screening with RHS-13 as advantageous compared to conventional methods for addressing mental health during the HE. However implementation requires implementation strategies to address known challenges related to language and time constraints (Robertshaw, Dhesi, and Jones 2017) and health care practitioners confidence in the tool’s effectiveness and value (Hagström et al. 2024).

## 7 Conclusion

This study highlights key factors associated with healthcare utilization among refugees and asylum seekers, including the association between mental health screening and increased access to care. Our findings show that individuals who completed screening were more likely to utilize healthcare services, underlining the importance of structured mental health assessments during. Recognizing and addressing mental illness in this group is crucial, as undiagnosed conditions can significantly affect employment, integration, and resettlement in the host country. Primary care and specifically the HE, can act as an entry point into mental health care services. The screening process itself likely serves as an intervention that legitimizes mental health concerns among refugees and asylum seekers. By formally addressing mental health status, using the RHS-13 may normalize help-seeking behaviors among the population as well as the professionals carrying out the screening. Future research should explore longitudinal approaches to better understand long-term healthcare utilization patterns and identify effective strategies to enhance access to mental health services for this population.

## Funding

The study has been funded by Stockholm Region, (grant 2018–0034, project 1513). The first author’s contribution was supported by Region Stockholm research funding (ALF). The funding did not influence the study design, data collection, analysis, interpretation of data or preparation of the manuscript. Open Access funding provided by Karolinska Institutet.

## Ethics declaration

### Informed consent and patient details

The authors have NOT obtained informed consent from participants or their legal representatives. Given the large number of individuals included in this study, retrospectively informing all registered individuals about the data processing conducted within this project would have constituted a disproportionate effort. In accordance with Article 14(5)(b) of the General Data Protection Regulation (GDPR), which provides an exemption from the duty to inform data subjects where doing so would involve disproportionate effort, individual notification was therefore not required.

### Studies in human

This study was performed in compliance with relevant laws, regulatory frameworks and guidelines where the research took place.

This study was approved by the Swedish Ethical Review Authority.

(Approval No. Dnr: 2019–01408).

## CRediT authorship contribution statement

**Sara Delilovic:** Writing – original draft, Project administration, Methodology, Investigation, Formal analysis, Conceptualization. **Anna Clara Hollander:** Writing – review & editing, Formal analysis, Conceptualization. **Knut Lönnroth:** Writing – review & editing, Conceptualization. **Henna Hasson:** Writing – review & editing, Funding acquisition, Formal analysis, Conceptualization.

## Declaration of competing interest

The authors declare that they have no known competing financial interests or personal relationships that could have appeared to influence the work reported in this paper.

## Data Availability

The data that supports the findings of this study are not directly available from the authors. Restrictions apply to the availability of these data, which were under license for the current study, and so are not publicly available. Data are available upon reasonable request and with permission of the Swedish Ethical Review Authority and Stockholm Region. For data request contact fouu.slso@regionstockholm.se.
